# Prospective Evaluation of Ventriculostomy Infections

**DOI:** 10.7759/cureus.312

**Published:** 2015-08-25

**Authors:** Emmagene Worley, Sonia Astle, Joe C Watson

**Affiliations:** 1 Department of Emergency Medicine, New York Presbyterian Hospital, University Hospital of Columbia and Cornell; 2 Department of Nursing, Inova Fairfax Hospital; 3 Department of Neurosurgery, Virginia Commonwealth University

**Keywords:** critical care, external ventricular drain, meningitis, ventriculitis

## Abstract

Introduction: Hospital-acquired infections associated with external drainage of ventricular cerebrospinal fluid (CSF) are a significant source of concern for the patients and the provider team alike. Traditional rates of ventriculostomy infection range from 10-17% in a time-dependent fashion. Changing physician and nursing practices fueled this concern over infections.

Objective: We sought to prospectively identify the risk factors associated with ventriculostomy infections as part of a quality assurance project.

Methods: One hundred consecutive patients were evaluated and data were collected on 91. The primary indications for ventriculostomy were subarachnoid hemorrhage (46%), intracerebral hemorrhage (24%), and trauma (22%). Variables prospectively evaluated included pre-incision antibiotics, sterile technique bundling, setting of placement (operating room versus intensive care unit), experience of operator (attending, resident, or physician assistant), catheter type (antibiotic impregnated or not), use of a post-insertion dressing, and in-dwell time of the catheter.

Results: There was only one infection in 91 patients (1.1%). This infection occurred in a patient without an antibiotic-impregnated catheter that was inserted by a resident physician. Compliance with pre-insertion antibiotics was very high, but most other variables had modest deviations in compliance.

Conclusion: Infection rate related to external ventricular drainage is very low. Our data suggest that non-antibiotic impregnated catheters may be associated with infection, but that other variables thought to be critical may be of less value.

## Introduction

Since the time of the first external ventricular puncture, credited to Wernicke in the late 1800s, infection control has remained a significant concern. Developments over the past decade with antibiotic-impregnated catheters, appropriate systemic antibiotic administration, and sterile technique have helped decrease the rate of post-ventriculostomy infection [1-3].

Ventriculostomies continue to be used for the management of increased intracranial pressure and hydrocephalus from a variety of etiologies, including subarachnoid, intracerebral or intraventricular hemorrhage, trauma, infection, or tumors [4]. This procedure, while life-saving, also carries significant complications, especially hemorrhage and infection. Of these, nosocomial infection related to external ventricular drainage catheters remains a potentially preventable cause of significant morbidity and mortality in patients with neurological illness [5-6].

The literature currently estimates ventriculostomy-associated infection incidence between 0% and 27% [4, 7-10]. Risk factors for infection include anything that provides a route for bacteria to enter the brain, such as craniotomies, skull fracture, lengthy duration of external drainage, irrigation, and frequent CSF sampling. Patient factors, such as systemic infection and intraventricular hemorrhage, also increase the risk for infection related to the ventriculostomy. These factors, which are inherent in the medically complex nature of these patients, complicate the relationship between ventriculostomies and infection [7-9].

Recently, there have been significant strides in technique and technology to reduce these nosocomial infections. Advances in the insertion technique over the last decade include needle tunneling, pre-administration of antibiotics, and impregnation of the catheters themselves with antibiotics. Furthermore, the development of standardized protocols and checklists has been thought to reduce infections throughout medicine and are used in this same manner for ventriculostomy placement [10-14].

In light of these advances, the infection rate traditionally quoted in the literature of up to 27% seemed high based on the authors’ clinical experiences. Therefore, we developed this prospective observational trial to determine 1) the ventriculostomy infection rate at our institution, 2) the risk factors associated with infection, and 3) if adherence to a strict technique and maintenance procedures would correlate with infections in this adult population. 

## Materials and methods

The Inova Health System Institutional Review Board issued approval for this study (#08.018). Informed patient consent was obtained prior to treatment.

One hundred consecutive patients in whom an external ventricular drain (ventriculostomy) was being placed were prospectively evaluated over a one-year period, from August 2008-August 2009, at Inova Fairfax Hospital, a large community hospital with a Level I trauma center. One hundred adults (> 18 years) patients were assessed and 91 enrolled in the study. Patients were included if they had a ventriculostomy catheter placed for intracranial pressure monitoring or drainage, were over 18 years old, and were a patient in the Emergency Department (ED), Operative Room (OR), Post-Anesthesia Care Unit (PACU), Trauma Intensive Care Unit (TICU), or Neurosurgical Intensive Care Unit (NSICU). Patients were excluded if the ventriculostomy catheter was placed at another hospital, had a known or recent history of meningitis or ventriculitis, or had a known positive CSF culture prior to insertion. All catheters were placed by physicians in the ED, OR, PACU, TICU, or NSICU. The procedure was observed by critical care nurses or neurosurgery physician assistants for adherence to sterile technique bundles and documented on the Data Collection Form (Appendix A); the variables monitored were grouped into bundles. The first bundle was the Insertion Bundle, including maximal barrier protection used at time of catheter insertion (cap and mask worn by anyone in the room, sterile gloves, sterile gown, full sterile body drape to cover patient’s head and body, and three minute Betadine Prep). The second bundle was the Maintenance Bundle, including mask/sterile gloves used when changing drainage bag, three minute prep with Betadine when changing the drainage bag, standardized specimen collection technique (three minute Betadine prep to collection port, mask, sterile gloves), and occlusive dry dressing, changed every 72 hours.

Data was also collected from critical care flow sheets, medical records, laboratory records on pre-procedure antibiotics, setting of placement, experience of operator, catheter type, use of a post-insertion dressing, and in-dwell time of the catheter. The patients were then followed until discharge for signs and symptoms of infection. Per protocol, CSF was sampled every three days or daily if the patient developed a fever (core temperature > 101.5° Fahrenheit). The primary endpoint of ventriculostomy infection was defined as a positive CSF culture after insertion.  Alternatively, ventriculitis could be defined by fulfilling the following clinical indicators: The patient has at least *one* of the following signs or symptoms with no other recognized cause: fever, headache, stiff neck, meningeal signs, AND at least *one* of the following: a) increased white cells, elevated protein, and/or decreased glucose in the CSF, b) organisms seen on Gram stain of CSF, c) organisms cultured from blood, d) positive antigen test of CSF, blood, or urine, or e) diagnostic single antibody titer (IgM) or fourfold increase in paired sera (IgG) for pathogen.

### Data analysis

Data was aggregated and analyzed with Premier *Quality Manager*, a risk-adjustment analytics program, for morbidity and mortality. Premier utilizes proprietary, disease-specific, risk-adjustment models developed from approximately 600 hospitals and 14 million patient records, to adjust for greater than 15 patient factors. The expected values generated reflect the top 15% of 2,700+ hospitals, nationally. Premier defines morbidity as the percentage of patients *estimated* to have at least one *serious complication *(having the greatest impact on resource intensity and clinical outcome)*.* The morbidity rate is estimated using probabilities assigned by physician panels (Project Osler). Pairings of principal and secondary diagnoses are assigned probability levels and multiplied for each patient to arrive at the overall probability of a patient having at least one serious complication. Protocol compliance data was analyzed with simple frequencies and percentages stratified in various ways to expose opportunities for compliance improvement.

## Results

### Patient characteristics

The median age of patients was 56 years. The primary diagnoses at the time of admission were subarachnoid hemorrhage (47%), intracerebral hemorrhage (24%), and secondary malignant neoplasm of the brain or spinal cord (5%). Aside from the ventriculostomy placement, other procedures included endovascular repair/occlusion head and neck vessels (17%), tumor resection (10%), and aneurysm clipping (7%). Eighty-five percent of patients presented via the emergency room. Most catheters were placed in the NSICU (58%), followed by the OR (24%). At the conclusion of care, patients were either discharged to home with either self-care or home health (33%), to inpatient rehabilitation (18%), to a skilled nursing facility (18%), or they expired (25%). The mortality rate was 24.7%, slightly higher but not statistically different from the expected mortality rate of 22.9%. The morbidity rate was 54.9%, also slightly higher but not statistically different from the expected morbidity of 50.5%.

### Ventriculostomy infection rate

Of the 91 patients who underwent catheter placement and monitoring, only one patient developed ventriculitis, confirmed with CSF samples as *Staphylococcus epidermidis* (1.1%). This infection occurred in a patient without an antibiotic impregnated catheter, placed by a resident physician.

### Compliance with technique bundles

Compliance with pre-insertion antibiotics was high. Pre-incision antibiotics were given in 71 out of 91 cases, with most cases (93%) receiving cefazolin. An antibiotic impregnated catheter (BACTISEAL^®^ EVD Catheter) was used in 91% of cases, and the non-antibiotic impregnated Integra catheter was used in the remaining 9% of cases.

Compliance with nursing care bundles, although somewhat variable, was also high. Overall bundle compliance, across all physicians and cases, was 88%. Administration of an iodine-based skin prep (3M™ DuraPrep™) evenly and the administration of antibiotics less than 60 minutes prior to insertion had the highest compliance rate at 98.9%. Post-procedure dressing had the lowest compliance at 70.3%. Compliance was high with sterile technique bundles, regardless of department, ranging from 50% compliance in two PACU cases to 95% compliance for 22 OR cases. Across all departments, hand hygiene had the highest compliance, while sterile dress application was most inconsistent: 85% of cases received sterile, dry dressing. 

## Discussion

We primarily set out to determine the ventriculostomy infection rate at our institution as a quality assurance project. With only one infection for 91 cases (1.1%), our infection rate is quite low. This correlates with our clinical experience and is much lower than previously stated in the literature. It demonstrates the effectiveness of new developments in infection control, including antibiotic impregnated catheters, antibiotic pre-administration, and standardization bundles. These advances combined to significantly lower the baseline infection rate.

Important strides in infection control have been in the area of antibiotic therapy, especially the development of antibiotic-impregnated catheters. In 2003, Zabramski, et al. reported in their prospective, randomized controlled trial that patients receiving the antibiotic-impregnated catheter were seven times less likely to develop positive CSF cultures compared with patients in the control group [10]. Then, a study by Harrop, et al., followed their institution’s infection rate as they introduced antibiotic impregnated catheters; the infection rate dropped from 8% to 1% [2]. However, once they discontinued their use of the antibiotic catheter secondary to high rates of occlusion, the infection rate immediately jumped back to their baseline of 8%. Resumption of a different, less occlusive, antibiotic-impregnated catheter decreased the infection rate again. While eight (8.8%) non-antibiotic impregnated catheters were used without infection in our study, the one infection that did occur was with a non-antibiotic impregnated catheter. This contributes to the literature, which suggests antibiotic impregnated catheters are protective against infection. The theoretical downside to these catheters lies in their propensity to change the microbial flora, selecting for antibiotic resistant bacteria. There is no evidence to date that susceptibility rates have changed, and antibiotic impregnated catheters remain a significant area of advancement in infection control.

Secondly, the routine use of antibiotic pre-administration has also helped to decrease surgical infection rates. In a study by Alleyne, et al., they compared the infection rate of long-term antibiotic patients to those who only received periprocedural antibiotics and found almost identical rates of infection, but significantly decreased cost [11]. The National Surgical Infection Prevention Project now recommends this periprocedural approach, administering antibiotics one hour prior to the procedure, and discontinuing them within 24 hours. In our study, the protocol dictated that antibiotics be given one hour prior to insertion, and compliance with this measure was one of the highest, at 93%.

The development of bundles and checklists has been thought to reduce infection and complication rates throughout medicine. Specific to ventriculostomies, Rahman, et al. at the University of Florida reduced infection rates from 9.2% to almost zero with the use of a standard protocol for ventriculostomy placement [14]. In addition, Korinek, et al. reduced their infection rate in Paris from 12.2% to 5.7% with standardization, and then, in their data analysis after implementation, found the most significant risks for infection were CSF leak and protocol violation [12]. Our study demonstrates fairly high compliance overall, with 88% adhering to the protocol, furthering the idea that standardization helps reduce infection rates. When broken down into its specific measures, there was much more variability in compliance rates, such as sterile dressing application with only 70% compliance. Infection rates did not increase for those patients, suggesting the sterile dressing itself is not as critical in infection prevention, but that sterile technique, as a whole, has more of an impact on infection risk. In fact, one hypothesis at the beginning stages of study design was that the lack of a sterile dressing would increase infection rates. The one infection occurred with a sterile dressing, but this study is not powerful enough to detect subtle correlations. It is clear, however, that sterile dressing application alone is not a significant risk or protective factor.

Our low rate of infection corroborates the opinion that our current techniques for ventriculostomy insertion and maintenance are an improvement over the past. One of our authors, JW, lends a personal perspective from a surgeon who has practiced through this era of change; he was a resident participant who placed catheters for the study by Holloway, et. al. in which there was a reported 10.4% infection rate in a multi-center study in traumatic brain injury [8]. He notes two primary differences in the insertion technique from then to now: 1) an improved tunneling technique using a trocar needle rather than a more traumatic technique that grabbed the catheter with a hemostat through a separate tunneling incision, and 2) antibiotic-impregnated catheters. 

The strengths and limitations of this study lie in its prospective, observational approach. With any observational study, behavior will change in response to being monitored. The rates of sterile technique compliance could be due to this subconscious effect on behavior. While this almost certainly occurred, we still believe this study accurately reflects real life behavior, given the persistently variable compliance rates. Based on only one infection, it is difficult to draw firm conclusions about the risk factors associated with these nosocomial infections. However, the efforts over the past decade appear to have been effective in decreasing infection incidence in ventriculostomy patients.

## Conclusions

Nosocomial infection from ventriculostomies remains an important concern, but new advances in antibiotic-impregnated catheters and standardization procedures have decreased this risk measurably compared to the historical literature. 

## Appendices

Ventriculostomy Catheter Study in Adult Patients

Ventriculostomy Insertion Bundle Compliance Data Collection Form

Date:                                       Time of insertion: 

Name of MD/PA inserting catheter: __________________________________________            

Form Completed By:_______________________________________________________

Location of insertion:  ED     NSICU     OR     TICU       Other:  ____________________

(please circle where the evd was inserted)

Type of Catheter:       Codman                       Integra                         Integra fiber optic

*(Circle which type)*          *(orange catheter)             (clear catheter)               (dual port)*

Compliance

Criteria

Y   N

Hand Hygiene prior to insertion

Y  N

Maximal barrier protection on insertion

Y  N

Betadine skin prep antisepsis

Y  N

Catheter insertion attempt:   one

Y  N

Occlusive dressing used following insertion

Y  N

Antibiotics administered prior to insertion

If yes, drug name, dose and time administered:

Name: 

Dose:

Length of time administered prior to procedure:

Y  N

Is catheter tunneled?

Definitions:

- Hand hygiene:  washed hands prior to gloves donning.
- Maximal barrier precautions:  includes use of cap, mask, sterile gown and gloves, full sterile body drape to cover patient’s head and body.  All in the room are required to wear masks.
- Betadine skin prep:  scrub catheter insertion site with Betadine solution using back and forth motion for three minutes prior to insertion.
- One catheter attempt:  was the insertion successful on the first pass?
- Occlusive dressing:  an occlusive dressing used to cover the insertion site.
- Catheter tunneled:  Catheter is inserted through one small incision/burr hole and the tubing connecting end of the catheter is tunneled under the scalp to exit through a second small incision in the scalp
